# Prevalence of *Toxocara* species infection in the U.S.: Results from the National Health and Nutrition Examination Survey, 2011-2014

**DOI:** 10.1371/journal.pntd.0005818

**Published:** 2017-07-31

**Authors:** Aaron Farmer, Thomas Beltran, Young Sammy Choi

**Affiliations:** 1 Infectious Disease Service, Department of Medicine, Womack Army Medical Center, Fort Bragg, North Carolina, United States of America; 2 Department of Clinical Investigation, Womack Army Medical Center, Fort Bragg, North Carolina, United States of America; 3 Departments of Medicine and Pediatrics, Womack Army Medical Center, Fort Bragg, North Carolina, United States of America; PUCRS, BRAZIL

## Abstract

Toxocariasis is one of the most common neglected infections of poverty in the U.S. with a reported National Health and Nutrition Examination Survey (NHANES) III (1988–1994) seroprevalence of 13.9% based on enzyme immunoassay testing. We reviewed NHANES data from 2011–2014 to assess current levels. Sera collected from NHANES 2011–2014 participants six years and older were tested for exposure using rTc-CTL-1 antigen, a more sensitive and specific recombinant antigen for IgG antibodies for *Toxocara* spp. These results were subdivided into children (age 6–17) and adults (age ≥ 18) and then compared between various sociodemographic characteristics. Given prior associations of *Toxocara* exposure with atopic disease and lead exposure, we also reviewed laboratory values including complete blood counts and blood and urine lead levels. Data from 13,509 individuals with *Toxocara* antibody results were examined including 3337 children (15.2%) and 10172 adults (84.8%). Overall seroprevalence was 5.1%. In adults increased antibody positivity occurred with non-White ethnicity, male gender, less than college-level education and lower income. Among children, increased antibody positivity was solely related to a lack of health insurance. Additionally, seropositivity was associated with increased blood lead and eosinophil levels in adults and both blood and urine lead levels in children. Relative to NHANES III (1988–1994), current data suggest an overall decrease in *Toxocara* spp. seroprevalence from 13.9% to 5.1%, however this may be artificially lowered due to difference in testing methods used. Persistent disparities appear to be associated with at-risk populations such as minority ethnicity and low socioeconomic status.

## Author summary

Toxocariasis is a pervasive helminth infection transmitted to humans via embryonated eggs from soil contaminated by the feces of dogs and cats (*Toxocara canis* and *T*. *cati*, respectively). The most recent seroprevalence study of *Toxocara* infection in the United States used the National Health and Nutrition Examination Survey (NHANES) III data collected from 1988 to 1994. At-risk groups identified included male gender, non-White ethnicity and low education and socioeconomic status. Since the original study, dogs and cats have increased in number and an improved testing platform has been created using rTc-CTL-1 antigen to *Toxocara* spp. with a sensitivity and specificity of 90% and 99%, respectively. In this study, we assessed current seroprevalence based on the NHANES data from 2011 to 2014 using the more specific testing platform. Although overall seroprevalence appears to have decreased from 13.9% to 5.1%; persistent disparities remain associated with at-risk populations including minority ethnicity and low socioeconomic status. Continued work is needed to improve awareness of disease transmission and treatment amongst the general population as well as veterinarians who may care for dogs and cats within the United States and take responsibility for education of the public.

## Introduction

Toxocariasis has been identified as the most common human parasitic worm infection in the world and one of the most common neglected infections of poverty in the U.S. [1–3]. Spread through contact with soil contaminated by embryonated eggs from dog and cat feces, the etiologic agents, *Toxocara canis* and *T*. *cati*, have classically been associated with two major clinical syndromes: visceral larva migrans and ocular larva migrans [4, 5]. Following accidental ingestion, *Toxocara* spp. larvae migrate to various organs including the lungs, liver and eyes producing an inflammatory response leading to manifestations of disease. Subclinical infection (also called “covert” or “common” toxocariasis) can occur, with some studies suggesting associations with decreased cognitive function, asthma and atopic disease [6–9].

As soil contamination with embryonated eggs can persist for years and remain infective, human exposure often results from contact with feces-contaminated soil from dogs and cats [10]. The potential for exposure is increased further through various reservoirs for *Toxocara* spp. including intestinal infections and somatic larvae in definitive hosts as well as larvae in paratenic hosts. There is also evidence of eggs found on the hair of definitive hosts such as dogs and cats which may then be transferred to their owners, albeit rarely [11]. Currently, control efforts are focused on removal of pet feces and covering sand pits in recreational areas such as parks and playgrounds and elimination of adult worms in these companion animals to limit risk of transmission from pets to humans [12–16].

To better define the extent of the problem, serological data from over 20,000 samples were tested via enzyme immunoassay in the Third National Health and Nutrition Examination Survey (NHANES III; 1988–1994) and revealed a seroprevalence of 13.9% for *Toxocara* antibodies [17]. Higher rates of antibody positivity were noted in children, non-Hispanic Blacks, low education/socioeconomic status and in those living in the South or Northeast areas of the U.S.; the primary influences were non-Hispanic Black ethnicity and county poverty [18]. Additional studies have also confirmed the disproportionate burden in those of lower education and socioeconomic status [2, 19, 20]. Given an increase in the numbers of dogs and cats of approximately 60 and 50%, respectively, from NHANES III (1988–1994) to prior to the current study time-period (2011–2014) as well as the availability of an improved testing platform using a Luminex based assay to rTc-CTL-1 antigen for *Toxocara* spp., we sought to use the latest NHANES data from 2011–2014 to reassess the seroprevalence of *Toxocara* antibody positivity in the U.S. as well as trends in exposure risk groups [17, 21–24].

## Materials and methods

### Study design and population

The NHANES is a series of ongoing cross-sectional surveys conducted by the Centers for Disease Control and Prevention (CDC) and is designed to assess the health and nutritional status of Americans through physical examinations and interviews [25]. Individuals in the survey participate in a household interview followed by physical examination in a mobile examination center. The NHANES sampling procedure is a complex multistage probability cluster design which oversamples specific populations such as Hispanics, non-Hispanic blacks, non-Hispanic Asians, older adults, and low income persons to obtain both adequate samples for meaningful subgroup analyses and more reliable parameter estimates [26].

The survey’s design and weighting methodology have previously been described [27]. To account for unequal selection probabilities among participants and adjustments for non-response, all estimates were weighted using multi-year sampling weights calculated from those provided by the National Center for Health Statistics (NCHS) to account for the two combined 2011–2012 and 2013–2014 NHANES cycles [28]. The data collection protocol was approved by the NCHS institutional review board.

### Demographic data and characteristics

Demographic information was obtained during the household interview. Self-reported socio-demographic characteristics included age at the time of the survey. Participants aged 6 to 17 were classified as children and those 18 years and older were classified as adults. Additional factors include gender, race/ethnicity (non-Hispanic white, non-Hispanic black, Hispanic, or other including multiracial), marital status (married or member of an unmarried couple; divorced, widowed, or separated; and never married), education level (did not graduate high school, graduated high school or attained a GED, some college or technical school, and graduated from college or technical school), health insurance coverage status, history of asthma, and family Poverty to Income Ratio (PIR). Pooling of demographic categories was conducted.

### Specimen collection and laboratory methods

Sera collected from NHANES 2011–2012 and 2013–2014 participants 6 years and older were tested for *Toxocara* spp. by a Luminex assay using recombinant rTc-CTL-1 antigen that detects IgG antibodies against *Toxocara* spp. The antibody test was classified as positive if the antibody responses to rTc-CTL-1 was greater than the cut-off point value of 23.1 mean fluorescence intensity [29, 30]. The assay itself has been validated against reference serum and has a sensitivity of 90% and a specificity of 99% [23].

### Statistical analysis

Analyses were conducted using NHANES provided statistical weights to account for the complex survey design. Weighted prevalence estimates are reported as percentages with 95% Wald confidence intervals (CI). Categorical variables were analyzed using Rao-Scott adjusted chi-square tests. Multivariable analysis was performed using logistic and linear regression models to determine predictors of *Toxocara* spp. infection. All statistical tests were performed by using a P < 0.05 level of significance. Data analyses were conducted using the complex sample package for SPSS 23 (IBM, Armonk, NY, USA).

### Ethics statement

Per NCHS standards, all adult participants provide written informed consent and for children <18 years of age, written informed consent by both the child and the parent/guardian was required. For this study, a waiver was obtained from the Womack Army Medical Center Institutional Review Board in accordance with the use of publicly available de-identified data.

## Results

A total of 13,509 individuals with valid *Toxocara* spp. antibody results participated in both the interview and examination portion of the 2011–2014 NHANES. The sample included 15.2% (N = 3,337) children and 84.8% (N = 10,172) adults. Overall, 858 (5.1%; 95% CI 4.4–5.9) participants tested positive for the *Toxocara* spp. antibody, representing over 12 million people in the greater non-institutionalized US population. Demographic and socioeconomic characteristics of children and adults are shown in Tables 1 and 2 respectively.

**Table 1:** Characteristics associated with *Toxocara* spp. infection in children.

		*Toxocara* spp. Positive		*Toxocara* spp. Negative		OR (95% CI)^1^
		n	% Estimate (95% CI)	n	% Estimate (95% CI)	
Sample Size		131	3.6 (2.7–4.7)	3206	96.4 (95.3–97.3)	
Age group, yrs						
	6–9	37	2.6 (1.6–4.0)	1112	97.4 (96.0–98.4)	NS^2^
	10–13	45	3.9 (2.7–5.6)	1079	96.1 (94.4–97.3)	
	14–17	49	4.1 (2.8–5.9)	1015	95.9 (94.1–97.2)	
Gender						
	Male	75	4.0 (2.8–5.5)	1644	96.0 (94.5–97.2)	NS^2^
	Female	56	3.2 (2.2–4.6)	1562	96.8 (95.4–97.8)	
Race/Ethnicity						
	Non-Hispanic white	29	3.1 (2.0–4.7)	804	95.9 (95.3–98.0)	NS^2^
	Non-Hispanic black	38	4.7 (3.2–6.8)	832	95.3 (93.2–96.8)	
	Hispanic	48	4.5 (2.8–7.0)	1061	95.5 (93.0–97.2)	
	Other race, including multicultural	16	2.7 (1.7–4.2)	509	97.3 (95.8–98.3)	
Health Insurance						
	Covered	107	3.2 (2.4–4.3)	2890	96.8 (95.7–97.6)	1 (Reference)
	No Health Insurance	23	7.3 (4.6–11.6)	313	92.7 (88.4–95.4)	2.4 (1.4–4.2)
Asthma History						
	Yes	26	3.4 (2.1–5.6)	631	96.6 (94.4–97.9)	NS^2^
	No	105	3.6 (2.7–4.8)	2574	96.4 (95.2–97.3)	
Ratio of family income to poverty^3^						
	Below federal poverty level	55	4.9 (3.1–7.7)	1034	95.1 (92.3–96.9)	NS^2^
	At or above federal poverty level	66	2.9 (2.1–4.2)	1985	97.1 (95.8–97.9)	

^1^ Odds Ratio based on regression analysis of 3333 participants with complete data.

^2^ NS: Non Significant.

^3^ Subsample does not sum to total sample due to non-responses.

**Table 2:** Characteristics associated with *Toxocara* spp. infection in adults.

		*Toxocara* spp. Positive		*Toxocara* spp. Negative		aOR (95% CI)^1^
		n	% Estimate (95% CI)	n	% Estimate (95% CI)	
Sample Size		727	5.3 (4.6–6.2)	9445	94.7 (93.8–95.4)	
Age group, yrs						
	18–29	107	4.4 (3.4–5.8)	2053	95.6 (94.2–96.6)	NS^2^
	30–39	92	4.8 (3.8–6.0)	1594	95.2 (94.0–96.2)	
	40–49	116	5.4 (4.3–6.8)	1529	94.6 (93.2–95.7)	
	50–59	130	6.4 (4.8–8.6)	1462	93.6 (91.4–95.2)	
	60+	282	5.7 (4.5–7.0)	2807	94.3 (93.0–95.5)	
Gender						
	Male	442	6.8 (5.9–7.9)	4489	93.2 (92.1–94.1)	1.9 (1.5–2.3)
	Female	285	4.0 (3.2–4.9)	4956	96.0 (95.1–96.8)	1 (Reference)
Race/Ethnicity						
	Non-Hispanic white	194	3.9 (3.1–4.9)	3945	96.1 (95.1–96.9)	1 (Reference)
	Non-Hispanic black	181	7.2 (5.8–8.8)	2063	92.8 (91.2–94.2)	1.5 (1.1–2.1)
	Hispanic	226	9.8 (7.6–12.6)	2001	90.2 (87.4–92.4)	1.6 (1.1–2.4)
	Other race, including multicultural	126	7.0 (5.7–8.7)	1436	93.0 (91.3–94.3)	1.8 (1.3–2.5)
Marital Status^3^						
	Married or living with partner	411	5.2 (4.4–6.1)	5209	94.8 (93.9–95.6)	
	Widowed, divorced, or separated	178	6.4 (5.0–8.0)	1940	93.6 (92.0–95.0)	NS^2^
	Never married	114	5.2 (3.9–6.9)	1793	94.8 (93.1–96.1)	
Education^3^						
	Less than high school graduate	279	11.5 (9.6–13.6)	1859	88.5 (86.4–90.4)	2.7 (1.9–3.7)
	High school graduate or GED	176	7.2 (5.9–8.8)	1891	92.8 (91.2–94.1)	2.1 (1.5–2.9)
	Some college	145	3.8 (2.9–4.9)	2830	96.2 (95.1–97.1)	NS^2^
	College graduate	103	2.9 (2.3–3.7)	2359	97.1 (96.3–97.7)	1 (Reference)
Health Insurance^3^						
	Covered	475	4.3 (3.7–5.0)	7410	95.7 (95.0–96.3)	1 (Reference)
	No Health Insurance	252	9.9 (7.9–12.2)	2024	90.1 (87.8–92.1)	1.5 (1.1–1.9)
Asthma History^3^						
	Yes	89	4.7 (3.6–6.0)	1469	95.3 (94.0–96.4)	NS^2^
	No	638	5.5 (4.7–6.4)	7968	94.5 (93.6–95.3)	
Ratio of family income to poverty^3^						
	Below federal poverty level	251	10.8 (8.9–13.0)	2034	89.2 (87.0–91.1)	1.8 (1.4–2.3)
	At or above federal poverty level	408	4.2 (3.6–4.8)	6685	95.8 (95.2–96.4)	1 (Reference)

^1^ Adjusted Odds Ratio based on multiple regression analysis of 8901 participants with complete data.

^2^ NS: Non Significant.

^3^ Subsample does not sum to total sample due to non-responses.

The prevalence of *Toxocara* spp. antibody for adults revealed no significant change in prevalence between the data collection cycles. In contrast, children experienced a significant reduction between 2011–2012 and 2013–2014 (P < 0.01). This was primarily associated with a decrease in prevalence among children with health insurance (P < 0.01, see Table 3); there was no difference in health care utilization.

**Table 3:** *Toxocara* spp. antibody prevalence by time.

		2011–2012				2013–2014	*P* ^1^
		n	% Estimate (95% CI)	n		% Estimate (95% CI)	
Children with Positive Antibody Result							
	With Health Insurance	81	4.5 (3.0–6.5)	26		1.7 (1.1–2.7)	<0.01
	Without Health Insurance	15	8.7 (5.0–14.8)	8		5.3 (2.1–12.4)	0.32
	Total	96	4.9 (3.4–7.0)	34		2.0 (1.4–2.9)	<0.01
Adults with Positive Antibody Result							
	With Health Insurance	248	4.6 (3.8–5.6)	227		4.0 (3.2–5.0)	0.30
	Without Health Insurance	142	10.5 (8.2–13.3)	110		9.1 (6.2–13.3)	0.54
	Total	390	5.8 (4.8–7.0)	337		4.9 (3.8–6.2)	0.27

^1^ P value based on Rao-Scott adjusted chi-square statistic.

Multivariable analysis of socio-demographic factors in adults identified the following risk factors for infection: gender (P < 0.001), race/ethnicity (P = 0.01), education (P < 0.001), healthcare coverage (P < 0.01), and PIR (P < 0.001). There was no significant difference between infection groups with regard to age category, marital status, or asthma diagnosis. Infection prevalence was higher among males compared to females and among non-White ethnicities. Additionally, the lack of health insurance and education was also associated with higher levels of infection among adults. Among children, only health insurance status distinguished seropositivity between the two groups (P < 0.01).

Table 4 shows laboratory results by *Toxocara* spp. seroprevalence and age category. Seropositive and seronegative children had a median blood lead concentrations of 0.98 (IQR 0.50–1.46) ug/dL and 0.53 (IQR 0.40–0.76) ug/dL, respectively (P < 0.01); median urinary lead concentrations were 0.50 (IQR 0.26–0.83) ug/dL and 0.26 (IQR 0.14–0.47) ug/dL, respectively (P < 0.01).

**Table 4:** Laboratory values by *Toxocara* antibody results.

		Toxocara Positive		Toxocara Negative		*P*^1^
		n	Median (IQR)	n	Median (IQR)	
Lead, urine (ug/L)						
	Adults	234	0.40 (0.20–0.66)	3082	0.33 (0.18–0.59)	0.37
	Children	37	0.50 (0.26–0.83)	1025	0.26 (0.14–0.47)	<0.01
Lead, blood (ug/dL)						
	Adults	560	1.24 (0.81–1.96)	7091	0.98 (0.63–1.54)	<0.01
	Children	120	0.98 (0.50–1.46)	2831	0.53 (0.40–0.76)	<0.01
Hemoglobin (g/dL)						
	Adults	727	14.40 (13.40–15.20)	9436	14.10 (13.20–15.10)	0.10
	Children	131	13.50 (12.90–14.50)	3204	13.50 (12.80–14.20)	0.75
Hematocrit (%)						
	Adults	727	42.20 (39.20–44.60)	9436	41.4 (38.70–44.20)	0.01
	Children	131	39.50 (37.60–42.10)	3204	39.40 (37.40–41.60)	0.90
RBC Folate (ng/mL)						
	Adults	390	411.00 (312.10–512.10)	4712	476.80 (359.40–618.10)	<0.001
	Children	97	4445.90 (312.10–525.40)	1663	450.30 (368.20–547.50)	0.18
TSH (mIU/L)						
	Adults	125	1.49 (1.16–2.00)	1563	1.58 (1.10–2.28)	0.16
	Children	16	1.16 (0.93–1.62)	252	1.49 (0.99–1.95)	0.08

^1^ P value based on adjusted Rao-Scott chi-square statistic.

Seropositive adults also had elevated serum lead levels compared to non-infected adults with a median of 1.24 (IQR 0.81–1.96) ug/dL vs. 0.98 (0.63–1.54) ug/dL (P <0.01), but urinary lead levels were not significantly different. Additionally, eosinophilia (defined as ≥ 500 eosinophils/uL) was present in 8.8% (95% CI, 6.1–12.5) of seropositive adults versus 5.2% (95% CI, 4.6–5.9) of seronegative adults (OR 1.7; 95% CI 1.2–2.5). No similar difference in eosinophilia was noted among children.

## Discussion

Relative to NHANES III (1988–1994), current U.S. data show an overall decrease in *Toxocara* spp. seroprevalence from 13.9% to 5.1%. This may be related to changes in exposure, a more specific testing platform, and perhaps modifications in veterinary practices and awareness [17, 31]. Seroprevalence remains elevated in certain groups such as minority ethnic groups, male gender and those with low education and socioeconomic status. Despite an overall decrease, this study of a nationally representative sample highlights the continued exposure of the U.S. population to this disease and the persistent disparities associated with at-risk populations.

Similar to the previous seroprevalence study utilizing data obtained over 20 years ago, we showed a persistent elevation in odds of antibody positivity in adults among minority ethnicity, male gender and low education level [17]. These findings overall mirror those found by Congdon et al. demonstrating minority ethnicity and poverty were important drivers of higher levels of *Toxocara* antibody positivity [8, 18]. Conversely, with non-Hispanic white as the reference, we found Hispanic and other race/multicultural ethnicities to have a higher risk of exposure than non-Hispanic Black. This is different than the studies by Won et al. and Congdon et al., which suggested lower rates of exposure in the Hispanic population [17, 18]. It has been hypothesized that the lower rates in Hispanic populations may be related to residence in more arid Western states with less likelihood for exposure to viable *Toxocara* eggs [19]. Unfortunately, no data were available in our study for region of the country or rural versus urban environment. Non-college level education (surrogate for lower socioeconomic status) and male gender have also previously been associated with increased *Toxocara* antibody positivity and were identified in this study as well [17, 32]. Although previously reported, age did not appear to be a risk factor for this cohort [17, 33].

For children, no difference was found in demographic data despite looking at multiple variables including age, gender, socioeconomic status and ethnicity. Additional factors that have previously been associated such as a diagnosis of asthma and eosinophilia were also not significantly different [8, 34]. Interestingly, a protective effect of health insurance was noted. Typically, antibody positivity might reflect lower socio-economic status, however in our study, a child’s family PIR was not a significant determinant of infectivity [35]. Furthermore, health care utilization rates did not differ between those with and without *Toxocara* infection.

From a testing standpoint, an additional consideration between NHANES III and the current data is the change in the testing platform itself. For NHANES III, seroprevalence was determined using *Toxocara canis* excretory secretory antigen enzyme immunoassay testing (TES-Ag EIA). Although this test has been the standard worldwide, the sensitivity is as low as 78% (for visceral larva migrans) and specificity has been limited by cross-reactivity, particularly in areas with other soil-transmitted helminthes such as *Ascaris* spp. [36]. Based on a review from 2014, prevalence of potential cross- reacting parasites such as *Ascaris* spp. may be as high as 49.4%, with higher rates in the southern U.S. and Appalachian regions [37]. Given this, results from prior seroprevalence studies may overestimate *Toxocara* exposure risk [1, 38]. For the most recent data, a newer assay based on rTc-CTL-1 antigen to *Toxocara* spp. was used with a sensitivity and specificity of 90% and 99%, respectively [23]. Thus, the current NHANES data likely is a better reflection of *Toxocara* exposure.

For the risk of exposure itself, the overall number of dogs and cats is an important factor. As mentioned previously, U.S. pet ownership has steadily increased from approximately 52 million dogs and 54 million cats at the time of NHANES III (1988–1994) to an estimated 70–83 million dogs and 74–96 million cats just prior to the current study time-period (2011–2014) [21, 22, 24]. As *Toxocara* spp. eggs are transmitted to the soil through dog and cat feces, the CDC encourages routine evaluation and treatment of house pets to limit human exposure as well as removal of any pet feces from recreational areas [39]. Household gardens have also been shown to have high levels of *Toxocara* ova, suggesting use of gloves during gardening activities may help decrease risk for exposure [40]. In addition, the Companion Animal Parasite Council suggests year round treatment for control of intestinal parasites [41]. At least one study suggests this therapy can reduce intestinal parasites by up to 91% in pet dogs when compared to shelter dogs, although there was no direct analysis of treated versus untreated dogs to rule out confounders such as decreased exposure between these two groups [31]. Prior studies have shown treatment must be provided at least four times per year for efficacy [42]. Unfortunately, as was demonstrated in a Dutch cohort of pet owners, as few as 16% of pet dogs and 24.5% of pet cats were at this goal; no similar studies were found in the U.S. [42, 43]. Additionally, in a study performed in Ireland, only 51.7% of pet owners believed pet feces could pose a risk to human health and only 4.3% had heard of *Toxocara* infection, potentially accounting for the low adherence to treatment recommendations [44]. With regards to prevention of environmental exposure, a study from Poland revealed continued high levels of soil contamination with *Toxocara* spp. eggs, particularly in backyards of rural and urban areas highlighting the fact that current preventive efforts may have limited efficacy; no similar studies were found in the U.S. [45].

Finally, as exposure to environmental lead and *Toxocara* spp. can both occur from exposure to contaminated soil, elevated lead levels are frequently found in *Toxocara* seropositive patients [17, 46]. In this study, blood lead levels showed a statistically significant difference between seropositive and seronegative adults. For adults, occupational exposure (particularly construction and manufacturing work) accounts for over 60% of lead exposure in those found to have very high blood levels [47]. As these occupations may be associated with increased dust, soil and outdoor exposure this may provide a link with infection. However, the data are conflicting on occupational exposure with at least one study of gardeners failing to show an increased risk whereas increased seroprevalence has been noted in waste pickers and those engaged in agricultural work [17, 20, 48, 49]. Occupational information was not available in the 2011–2014 cycle so further comparison could not be made in this study. It is also important to note that while statistically significant differences occurred, blood lead levels noted in those seropositive were considerably less than the CDC reference value of 5 ug/dL.

Other laboratory findings in adults included a lower RBC folate (P < 0.001) and higher eosinophil count (P < 0.01) in seropositive samples. Review of the hemoglobin and hematocrit levels between seropositive and seronegative adults did not differ so the significance of a lower RBC folate is likely negligible. Also, although *Toxocara* infection has been associated with atopic diseases (such as asthma) and eosinophilia, no difference was noted for asthma and the absolute difference in eosinophil count noted was minimal and thus not likely to be clinically significant [50, 51].

For children, blood and urine lead levels were elevated in those with *Toxocara* antibody positivity, which has been identified in previous studies [7, 17]. The concurrent elevation of both *Toxocara* antibody and lead levels is concerning as both have been independently associated with decreased cognitive function in children [7, 17, 52]. In our study, the mean serum level of 1.43 ug/dl in seropositive children, though statistically higher than those seronegative, was well below the CDC reference level of 5 ug/dL. Despite this cut-off, no “safe” level of blood lead has been identified in children and thus interventions aimed at decreasing the common route of exposure (i.e. geophagy) may help decrease rates of both [53–55].

Several limitations of this study should be noted. This study did not include children younger than 6 years old. Also, we could not assess the extent to which higher seroprevalence might have reflected birth, prior residence outside the U.S. or current residence. In addition, antibody testing does not distinguish between current and prior infection so higher rates in adults may be related to antibody persistence from infection at a younger age. Although little research has been done, most studies show that antibody positivity tends to wane with time and thus this is unlikely to have impacted our findings [56]. Finally, the use of a different testing platform with increased *Toxocara* sensitivity and specificity may limit the ability to make comparisons between prior reports and current data.

In conclusion, *Toxocara* spp. continues to affect a considerable portion of the U.S. non-institutionalized population and risk for exposure may increase as dogs and cats enter more homes. Significant gaps in research remain, particularly for *Toxocara* spp. contamination in various areas of the U.S. such as public parks, recreational areas and urban vs. rural locales. Studies assessing current CDC recommendations in reducing exposure such as disposal of pet waste and hand washing are also limited. Infection could be decreased with improved awareness in both the general population and U.S. veterinarians of exposure risks and knowledge of the effectiveness of anthelminthic treatment of dogs and cats.

## Supporting information

S1 Checklist STROBE checklist. (PDF)
